# Editorial: Emerging Roles of TRP Channels in Brain Pathology

**DOI:** 10.3389/fcell.2021.705196

**Published:** 2021-06-07

**Authors:** Bilal Çiğ, Sandra Derouiche, Lin-Hua Jiang

**Affiliations:** ^1^Department of Physiology, Faculty of Medicine, Ahi Evran University, Kirşehir, Turkey; ^2^Division of Cell Signaling, National Institute for Physiological Sciences, Okazaki, Japan; ^3^Faculty of Biological Sciences, School of Biomedical Sciences, University of Leeds, Leeds, United Kingdom

**Keywords:** TRP channels, ischemic stroke, neurodegenerative diseases, psychiatric disorders, glioma, Ca^2+^ signalling

The mammalian transient receptor potential (TRP) ion channel superfamily comprises six subfamilies, TRPC (canonical), TRPV (vanilloid), TRPM (melastatin), TRPA (ankyrin), TRPP (polycystin), and TRPML (mucolipin) (Ramsey et al., 2006; Venkatachalam and Montell, 2007). TRP channels are tetrameric and each subunit contains intracellular N- and C-termini and six membrane-spanning segments, with the fifth and sixth segments and the re-entrant loop between them forming the ion-conducting pore (Cao, 2020). They function as non-selective cation channels, with prominent Ca^2+^ permeability for most of them, and are activated by diverse physical, chemical and biological stimuli. Their Ca^2+^ permeability, poly-modal activation and wide expression place these channels in a vital position mediating Ca^2+^ signalling in a range of physiological processes. Not surprisingly, accumulating evidence supports an important role for the TRP channels in the pathogenesis of numerous diseases (Nilius et al., 2007). Many TRP channels are expressed in the brain. This Research Topic, including 14 review and original research articles, offers critical and new insights into the role of TRP channels, particularly the Ca^2+^-permeable ones, in multiple brain pathologies.

## Trpc in Ischemic Brain Damage

Brain is highly vulnerable to damage by ischemia, if severe or lasting, and reperfusion after transient ischemia. Jeon et al. have critically evaluated the literature and also presented their recent study regarding the role of TRPC channels in ischemic brain damage. A complex role for the TRPC channels is emerging from studies, using middle carotid artery occlusion followed by reperfusion (MCAO/R), an *in vivo* model of ischemic stroke, and neuronal death induced by oxygen and glucose deprivation followed by reoxygenation (OGD/R), an *in vitro* model of ischemia/reperfusion, in combination with using transgenic knockout (KO) mice and neurons or brain slices derived from KO mice or using pharmacological interventions. TRPC channels, including TRPC3, TRPC4, TRPC6, and TRPC7, play a significant role in mediating ischemic brain damage. TRPC1 was proposed to act as a protective mechanism against ischemic brain damage via inhibiting generation of reactive oxygen species (ROS). However, Jeon et al. showed that TRPC1-KO attenuated OGD/R-induced neuronal death.

Neuroinflammation is another mechanism for ischemic brain damage and many other brain pathologies. Liu et al. showed that inhibition or depletion of TRPC6 suppressed OGD/R-induced Ca^2+^ response, production of interleukin (IL)-1β and IL-6, two neurotoxic proinflammatory cytokines, caspase-3 activation and apoptosis in astrocytes. Inhibition of TRPC6 also attenuated MCAO/R-induced caspase-3 activation, elevated level of IL-1β and IL-6 in the peri-infarct areas, and infarction in mice. These results support a critical role for TRPC6 in astrocytes in mediating neuroinflammation and ischemic brain damage.

## Trp in Neurodegenerative and Neurological Diseases

Alzheimer's disease (AD), Parkinson's diseases (PD), Huntington's disease, amyotrophic lateral sclerosis and epilepsy represent the most common neurodegenerative and neurological diseases. Extensive research efforts have been devoted to exploring the TRP channels in the pathogenesis of these conditions. Lee et al. have provided a concise overview of the potential involvement of the TRP channels in AD (TRPC1, TRPC6, TRPV, TRPM2, TRPM7, and TRPML1), PD (TRPC1, TRPC4, TRPC5, TRPM7, and TRPML1), Huntington's disease (TRPC1 and TRPC5), amyotrophic lateral sclerosis (TRPC4, TRPM2, TRPM3, TRPM7, and TRPML1) and epilepsy (TRPC4, TRPC5, TRPV4, TRPM7, and TRPA1). They further elaborated diverse TRP-mediated Ca^2+^-dependent downstream signalling pathways to the associated pathologies. Vaidya and Sharma have drawn their attention to the TRP channels expressed in substantial nigra pars compacta and other brain areas affected in PD. TRPC1 was shown to protect PD-related neuronal death. Intriguingly, both activation and inhibition of TRPV1, reported by different studies, improved motor function through regulation of distinctive molecular and cellular mechanisms. Oxidative stress, due to accumulation of high levels of ROS, is a pathological factor as well as a conspicuous pathological feature in PD (Prasad and Hung, 2020). Vaidya and Sharma also discussed the role of TRPM2 and TRPM7, both known to be sensitive to activation by ROS, in mediating PD-related neuronal death.

## Trpv in Bbb Dysfunction and Related Brain Pathologies

Endothelial cells play a key role in forming blood brain barrier (BBB), and BBB dysfunction results in infiltration of peripheral immune cells into the brain to exacerbate neuroinflammation and thereby ischemic stroke and neurodegenerative diseases (Sweeney et al., 2018). TRPV4 is known to regulate endothelial barrier function via mediating Ca^2+^ influx into endothelial cells. Consistently, Rosenkranz et al. demonstrated that pharmacological inhibition of TRPV4 in mouse brain microvascular endothelial cells improved endothelial barrier function. Such an effect was however obliterated in endothelial cells after exposure to interferon-γ and tumour necrosis factor-α that down-regulated the TRPV4 expression. In mice, TRPV4-KO failed to prevent BBB dysfunction and associated experimental autoimmune encephalomyelitis, a model of multiple sclerosis, or MCAO/R-induced brain damage, suggesting loss of TRPV4-mediated regulation of BBB function under inflammation. Luo et al. examined and revealed a different profile of TRPV1-4 expression in brain endothelial cells of rat and human origins, ranking from high to low: TRPV4 > TRPV2 > TRPV3 > TRPV1 in rat endothelial cells, and TRPV2 >> TRPV4 > TRPV1 > TRPV3 in human endothelial cells. Thus, TRPV4 and TRPV2 represent the predominant TRPV in human and rat brain endothelial cells, respectively. Such species difference may complicate testing drugs using rat models of BBB dysfunction and related brain pathologies.

## Trpm3 in Developmental Disorders

TRPM3 is activated by endogenous neurosteroid pregnenolone sulphate. TRPM3 expression is documented in several brain regions, including hippocampus and choroid plexus, and in cerebellar Purkinje neurons and oligodendrocytes. Held and Tóth have reviewed the potential role of TRPM3 in brain physiological and pathophysiological functions, and highlighted genetic alterations in the TRPM3 gene in patients with development and intellectual disabilities. Particularly interesting is the identification of *de novo* gain-of-function mutations in patients with developmental and epileptic encephalopathies. Deletions in the TRPM3 gene were found in patients with Kabuki syndrome, a multisystem disorder including intellectual disability, and in autism patients. However, it remains unclear how alteration of TRPM3 affects brain development and other functions.

## Trp in Psychiatric Disorders

Psychiatric disorders such as depression and anxiety disorders are caused by diverse and complex factors, both genetic and environmental. As proposed in neurodegenerative diseases, oxidative stress is a critical pathological factor, and Ca^2+^ signalling is disrupted, in psychiatric disorders. Nakao et al. have overviewed the redox-sensitive Ca^2+^-permeable TRP channels in neurons and glial cells, including TRPC4, TRPC5, TRPV1, TRPM2, and TRPA1, in regulating cell functions. They have provided a concise summary of the studies that showed using a battery of behaviour tests that anxiety-like behaviours in mice were attenuated by genetic deletion (TRPC4, TRPC5, TRPV1, TRPM2, or TRPA1) or pharmacological inhibition (TRPC4/TRPC5). They also discussed the possible mechanisms by which these channels mediate oxidative stress-induced Ca^2+^ signalling and subsequent alterations in neuronal connectivity, synaptic plasticity and glial cell function, leading to psychiatric disorders.

## Trp in Chronic Pain and Its Inhibition by Resolvins

Chronic pain occurs as a result of neuronal tissue inflammation and/or damage. Increased expression and/or activation of TRP channels in nociceptive neurons can enhance the excitability of nociceptive neurons and thereby intensify the pain signals. Resolvins are a class of lipid mediators generated during the resolution phase of acute inflammation. Roh et al. discussed the studies showing analgesic effects of different resolvins against inflammatory pain, through activating distinct cognate receptors to inhibit nociceptive TRP channels, including TRPV1, TRPV3, TRPV4, and TRPA1. The role of such inhibitory mechanisms in neuropathic pain is unknown.

## Trp in Brain Tumours

Malignant glioma including glioblastoma is the most common group of primary brain tumours and exhibits resistance to treatment and high recurrence. Chinigò et al. have conveyed the literature informing the expression of TRP channels and their potential role in brain tumours. The expression in glioma tissues and cells was shown to be upregulated (TRPC1, TRPC6, TRPV4, TRPM2, TRPM7, TRPM8, and TRPML2), or down-regulated (TRPV1, TRPV2, and TRPML1). Such alterations in some cases exhibited correlations with glioma progression and overall patient survival. Moreover, some of these channels (TRPC1, TRPC6, TRPV4, TRPM7, TRPM8, and TRPML2) appear pro-tumorigenic and, conversely, others (TRPV1, TRPV2, TRPM2, TRPM3, TRPA1, and TRPML1) are anti-tumorigenic. Chinigò et al. have proposed diverse signalling pathways, downstream of TRP channel-mediated elevation in intracellular Ca^2+^, in the regulation of glioma cell proliferation, migration and invasion as well as cell death.

## Trp in Regulating Ca^2+^ and H^+^ Homeostasis and Interacting With Other Signalling Mechanisms

Disruption in Ca^2+^ homeostasis has been alluded by multiple articles as an important mechanism in the pathogenesis of brain pathologies. Plasma membrane Ca^2+^-ATPase drives Ca^2+^ efflux and H^+^ influx and thus plays a vital role in modulating intracellular Ca^2+^ and H^+^ homeostasis. Hwang et al. have reviewed the role in neurodegenerative diseases of the plasma membrane Ca^2+^-ATPase in concerted actions with Ca^2+^-permeable TRP channels in altering Ca^2+^ and H^+^ homeostasis in neuronal and glial cells.

Hermann et al. investigated contribution of dinucleotide nicotinic acid adenine phosphate (NAADP)-induced Ca^2+^ release in mouse hippocampal neurons to the Ca^2+^ response evoked by glutamate, the major excitatory neurotransmitter in the brain. They proposed that NAADP induces Ca^2+^ release from intracellular acidic stores by activating TRPML1 and two-pore channels, and also from ER by activating ryanodine receptors, and showed that inhibition of NAADP signalling reduced glutamate-evoked Ca^2+^ response, particularly the sustained component. The study demonstrated substantial contribution of NAADP to glutamate-induced Ca^2+^ signalling in hippocampal neurons.

The activity of small Rho GTPases is critical for actin-based cytoskeletal remodelling. Ca^2+^ is an intracellular signal upstream of Rho GTPases. Lavanderos et al. have introduced the role of Rho GTPases in the regulation of axon growth, dendritic spine development, synapse formation, glial cell migration, and endothelial permeability, which are vital in sustaining brain structure and function. They further presented the potential Rho GTPase-dependent mechanisms that mediate Ca^2+^-permeable TRP channels in various brain conditions, including AD (TRPC6 and TRPV1), ischemic bran damage (TRPM7), glioma and neuroblastoma (TRPC6, TRPV1, TRPM7, and TRPM8).

Manchanda et al. extended their previous work to study TRPV1 in regulating the generation of endocannabinoids. Treatment of human embryonic kidney 293 cells expressing TRPV1 with capsaicin increased the levels of 2-acyl glycerols and reduced the levels of N-acyl ethanolamines, depending upon temperature. These results support the interesting notion that the TRPV1 and the receptor for endocannabinoids cross-talk in response to changes in temperature.

Collectively, the insightful evaluation of the literature and the new information presented in this Research Topic have evolved a better understanding of the TRP channels in the pathogenesis of multiple brain pathologies, and also raised many outstanding questions that need further researches in order to gain comprehensive and mechanistic insights into these pathological conditions and provide the proof of concept of targeting the TRP channels and/or related mechanisms as potential therapeutic strategies.

## Author Contributions

All authors have contributed to the writing, the revision of this Editorial Article, and approved it for publication.

## Conflict of Interest

The authors declare that the research was conducted in the absence of any commercial or financial relationships that could be construed as a potential conflict of interest.
